# Non-inflammatory Physiology of “Inflammatory” Mediators – *Unalamation*, a New Paradigm

**DOI:** 10.3389/fimmu.2020.580117

**Published:** 2020-10-07

**Authors:** Krishna Rao Maddipati

**Affiliations:** Department of Pathology, Wayne State University, Detroit, MI, United States

**Keywords:** parturition, polyunsaturated fatty acids, specialized pro-resolving lipid mediator (SPM), NSAID (non-steroidal anti-inflammatory drug), prostagladin E(2), epoxygenase, cyclooxyenase-2, inflammation

## Abstract

Many small molecules (mostly lipids derived from polyunsaturated fatty acids) and proteins (e. g., cytokines and chemokines) are labeled as inflammatory mediators for their role in eliciting physiological responses to injury. While acute inflammatory events are controlled by anti-inflammatory drugs, lasting damage to the tissues as a result of persistent inflammation is increasingly viewed as the root cause of many chronic diseases that include cardiovascular, neurological, and metabolic disorders, rheumatoid arthritis, and cancer. Interestingly, some of the “inflammatory” mediators also participate in normal developmental physiology without eliciting inflammation. Anti-inflammatory drugs that target the biosynthesis of these mediators are too indiscriminate to distinguish their two divergent physiological roles. A more precise definition of these two physiological processes partaken by the “inflammatory” mediators is warranted to identify their differences. The new paradigm is named “*unalamation*” ('ə‘*n'*ə*lAmāSH(*ə*)n*) to distinguish from inflammation and to identify appropriate intervention strategies to mitigate inflammation associated pathophysiology without affecting the normal developmental physiology.

## Lipid Mediators of Inflammation

Inflammation is a physiological response of the biological system to injury. The injury can either be sterile (physical injury or stress) or pathogen driven. Clinically, inflammation is still defined by the original description of Celsus in the first century of CE with four cardinal signs, *viz*. redness, swelling, pain, and heat as well as loss of function of an organ, later added by Galen (1). The biochemical pathways that lead injury to inflammatory response are highly defined, even if not fully elucidated. Inflammation is biochemically characterized by local increase in the concentration of lipid mediators such as prostaglandins, leukotrienes, and platelet activating factors, leukocyte derived reactive oxygen species as well as protein mediators such as cytokines, adenosine receptors, and cell adhesion molecules (2). The lipid mediators are biosynthesized *ad hoc* at the site of injury through a complex cascade of receptor activation, gene expression, substrate mobilization, and enzyme reactions, whereas the protein mediators are released from intracellular stores in response to injury or infection. The invariable association between inflammation and these lipids (as well as cytokines) is so strong that they are labeled as “mediators of inflammation,” for their chemotactic properties and to elicit response from immune cells. Inflammation that serves the purpose of clearing the pathogenic microbes or dead cells resulting from an injury with the aid of strong oxidants generated by neutrophils and macrophages also results in incidental damage in the surrounding tissue, manifesting in the observed clinical symptoms. Waning of inflammation, i.e., resolution, attempts to return the system to its normal (homeostatic) state. Resolution of inflammation was once thought to be passive by way of dissipation of inflammatory mediators through simple dilution in circulation or metabolism. However, it is now recognized as an active process involving an alternate set of lipid mediators mostly derived from ω-3 polyunsaturated fatty acids (with the exception of lipoxins) such as resolvins, protectins, and maresins (3). The current view is that peak of inflammation is implicitly followed by an active resolution phase to return the system to homeostasis. Any delay or lapse in the initiation of resolution could lead to an exacerbated state of inflammation and failure of the system to return to homeostasis (4). Persistent activation of adaptive immune response by lymphocytes, macrophages, and plasma cells resulting from an inadequate or incomplete resolution of inflammation leads to its chronicity and is increasingly viewed as an essential contributor to the etiology of all chronic diseases (5, 6).

**Figure 1:**
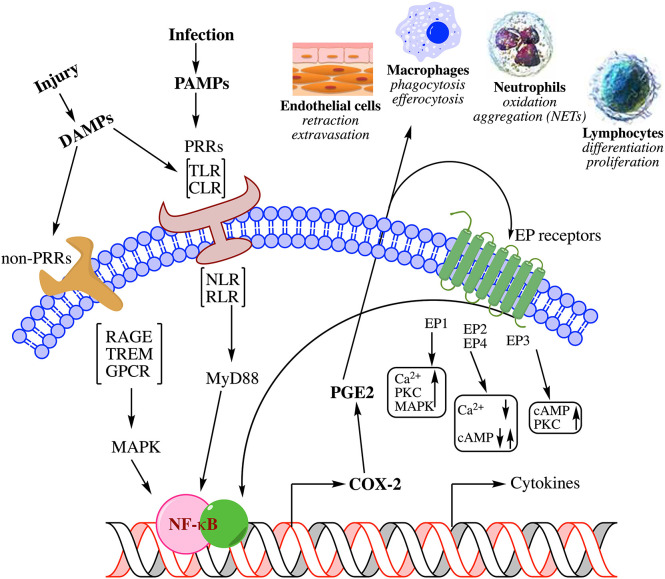
Summary of biochemical pathways leading to inflammation in response to injury. Physical or pathogen driven injuries are perceived by Pattern Recognition Receptors [PRR; e.g., Toll-Like Receptors (TLR), C-type Lectin Receptors (CLR), Nucleotide-binding Oligomerization Domain (NOD)-Like Receptors (NLR), Retinoic acid-Inducible Gene (RIG)-I-Like Receptor (RLR)] as well as non-PRRs [e.g., Receptor for Activated Glycated End products (RAGE), Triggering Receptors Expressed on Myeloid cells (TREM), G-Protein Coupled Receptors (GPCR)], whose ligands include damaged cell debris (DAMP) of the host or invading pathogen (PAMP). These receptors activate transcription factors such as Nuclear Factor kappa-light-chain-enhancer of activated B cells (NF-κB) *via* adapter proteins like Myeloid Differentiation 88 (MyD88) and Mitogen-Activated Protein Kinase (MAPK) that induce the biosynthesis of lipid mediators such as prostaglandin E_2_ (PGE_2_) through cyclooxygenase-2 (COX-2) induction and release of cytokines. These mediators mutually amplify their response both through autocrine and paracrine activation of specific receptors [receptors (EP) for PGE_2_ shown here] as well as induce endothelial retraction and extravasation, phagocytosis and efferocytosis by macrophages, oxidative metabolism of pathogens by neutrophils, and induction of immune response by lymphocyte differentiation; all manifest in the clinical symptoms of inflammation. For a detailed review on the biochemistry of inflammation and the role of lipid mediators please see these comprehensive reviews (7–10).

**Figure 2:**
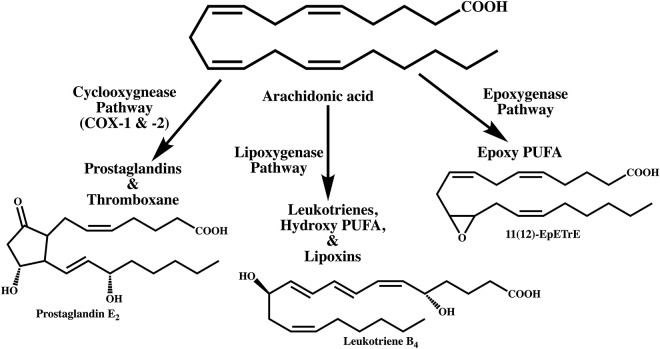
Metabolism of arachidonic acid by three different pathways leading to the biosynthesis of lipid mediators that participate in inflammation. Prostaglandin E_2_ is the major metabolite of the cyclooxygenase pathway. Of the three major lipoxygenases that metabolize polyunsaturated fatty acids, leukotriene B_4_ is biosynthesized from the 5-lipoxygenase pathway. The epoxygenase pathway, catalyzed by cytochrome P450 enzymes generates arachidonic acid epoxides. The epoxygenases are less regiospecific but each isoenzyme is known to catalyze the biosynthesis of one major epoxide. Only the 11(12)-epoxyeicosatrienoic acid (11(12)-EpETrE) is shown as an example.

Of particular importance to the initiation of inflammation are lipid mediators, since they are generated first at the site of injury to elicit an immunological response (Figure 1). Lipid mediators, especially those derived from the metabolism of polyunsaturated fatty acids (PUFA), are biosynthesized locally in response to injury by a cascade of biochemical events starting from the release of PUFA from phospholipids (Figure 2) (11)^1^. PUFA are metabolized by cyclooxygenases (also known as prostaglandin H synthases, PGHS or COX) to prostaglandins (e.g., PGE_2_) and by lipoxygenases to leukotrienes (e.g., LTB_4_) that serve as autocrine lipid mediators of inflammation (12). There are two isoforms of the cyclooxygenase, COX-1 and -2, that initiate the biosynthesis of prostaglandins. Of these, COX-1 is constitutively expressed in most tissues. Among the lipid mediators generated in the cyclooxygenase pathway of arachidonic acid metabolism, PGE_2_ exhibits the most pleotropic biological activities (7, 13–15). These include both homeostatic and pathophysiological functions regulating such diverse activities as blood pressure, gastrointestinal integrity, fertility, as well as inflammatory response (16). The fact that PGE_2_ is ubiquitously generated in every tissue adds to the conundrum of diametrically opposing biological activities. PGE_2_ participates in every process leading up the clinical manifestation of inflammation. Until the discovery of the second isoform, COX (which was later designated as COX-1) and prostaglandins (in addition to leukotrienes) generated by it from arachidonic acid were the default lipid mediators of inflammation. The discovery of a second, inflammatory stimuli-inducible COX (COX-2) shifted the focus to prostaglandins generated by this new enzyme as lipid mediators of inflammation as well as the target of non-steroidal anti-inflammatory drug development (17–19). It is now well-established that COX-1 participates in normal homeostatic physiology (20). So is the recognition of the inducible COX-2 as an inflammatory enzyme. However, constitutive expression of COX-2 is also observed in human tracheal epithelial cells, gastric mucosa, cardiomyocytes, brain, and kidney along with select cells in myriad other tissues (21–28). While the precise sequence of events is still the subject of active research, induction of COX-2 to initiate the biosynthesis of prostaglandins is one of the earliest steps in the events leading to the clinical manifestation of inflammation, suggesting that induction (not necessarily the constitutive expression) of COX-2 and the resulting biosynthesis of PGE_2_ are synonymous with inflammation (29). However, examples are abound where COX-2 induction and PGE_2_ biosynthesis do not lead to inflammation by default. Thus, the very idea of COX-2 induction as inflammatory is highly contextual and defining a new physiological process without any connotations of inflammation is vital to our understanding of developmental physiology mediated by the very same molecules. The proposed new concept has broader implications on inflammation and developmental physiology.

## Non-Inflammatory Physiology of Prostaglandins and Cytokines in Parturition

A physiological process where prostaglandins play an essential role is human parturition. Intra-amniotic concentrations of PGE_2_ and PGF_2_α are rapidly increased, within hours, leading up to the initiation of labor at term (30). Median amniotic fluid concentrations of PGE_2_ are about 24 nM at term before the onset of labor and increase nearly 3-fold−65 nM with the onset of labor (31, 32). This increase is a result of increased expression of COX-2, unique to spontaneous labor at term in the reflective amnion epithelial cells (33, 34). In addition to the prostaglandins, inflammatory cytokines (IL-1a, -1b, -2, -6, -7, -12, -15, -16, -17, and -23, TNF-α and -β, IFN-γ, GM-CSF) and anti-inflammatory cytokine IL-10 are increased 4-5-fold in spontaneous labor at term (35, 36). EP and FP receptors that mediate the physiological effects of PGE_2_ and PGF_2_α, respectively, are also expressed in pregnant myometrium and their absence leads to a failure of parturition (37–39). Thus, the strong association between COX-2 induction and inflammation led to the assumption that parturition, where significant COX-2 induction is observed in placental membranes and myometrium (33, 34, 40), is an inflammatory event (41–43). However, except for the pain associated with uterine contractions (and not due to edema and diapedesis), other cardinal signs of inflammation (especially fever) are not manifested in parturition, except in clinical chorioamnionitis (36, 44, 45).

Clinical chorioamnionitis is an inflammatory condition of the pregnant woman that is characterized by maternal fever, maternal and/or fetal tachycardia, leukocytosis, uterine tenderness, and foul-smelling amniotic fluid (36). Biochemically, this inflammatory condition is characterized by elevated levels of intra-amniotic IL-6 (>2.6 ng/ml) and neutrophil infiltration of the intra-amniotic space (46). While it is long believed that microbial invasion of the amniotic cavity is responsible for clinical chorioamnionitis, recent evidence shows that intra-amniotic infection accounts only for 54% of the cases (47). Of the remaining 46% of the cases with this inflammatory condition, 24% have no detectable microbial invasion and 22% have neither microbial invasion nor elevation of IL-6 in the amniotic fluid or neutrophil infiltration. Considering the induction of biosynthesis of the inflammatory lipid mediators such as PGE_2_ and PGF_2_α in human amniotic fluid at term in spontaneous labor, it would be tempting to speculate a further increase in the levels of these inflammatory lipids as responsible for clinical chorioamnionitis (despite the fact that concentrations of PGE_2_ would be at the EP receptor-saturating levels even in uneventful spontaneous labor at term). However, lipidomic analysis of human amniotic fluid shows no significant differences in the amniotic fluid levels of PGE_2_ or PGF_2_α (or their downstream metabolites) between patients in spontaneous labor with or without clinical chorioamnionitis, including patients with demonstrable microbial invasion of the amniotic cavity (48).

## “Inflammatory” Lipid Mediator and Receptor Sufficiency, Yet no Inflammation

Biological activities of prostaglandins are mediated by highly selective and cell-specific expression of G-protein coupled receptors. For PGE_2_, there are four receptor subtypes (EP1-EP4) expressed in a tissue-specific manner (49). EP receptor signaling is mediated by second messengers such as cAMP, Ca^2+^, and IP3 to elicit an inflammatory response by inducing vascular permeability, neutrophil extravasation, and hyperalgesia (50). Spatiotemporal expression of EP receptor subtypes in human placental membranes and myometrium illustrates their unique physiological roles (38, 39). For example, expression of EP2 receptor (smooth muscle relaxant receptors) in myometrium is maintained throughout the pregnancy, contributing to quiescence, but reduced at term in labor. EP3 receptors (smooth muscle contractile receptors), on the other hand, are increased leading to labor at term and mobilized more toward the lower segment myometrium, contributing to smooth muscle contractions during labor (38, 51, 52). The binding constant for PGE_2_ to EP3 receptors is about 1 nM (53). Considering the fact that the human amniotic cavity is an isolated and privileged space, the amniotic fluid concentrations of PGE_2_ (median 65 nM at term in spontaneous labor) are true local concentrations of this inflammatory mediator in which the fetus bathes for several hours before delivery. PGE_2_ is an immune modulator as well as endogenous pyrogen and EP3 receptor activation is an established physiological event in fever induction, one of the classical symptoms of inflammation (8, 54, 55). Therefore, there is no dearth of prostaglandin receptors or their ligands in uteroplacental tissues, yet, there is no fever associated with spontaneous parturition at term, except in case of clinical chorioamnionitis.

## Inflammatory Mediators Elicited by Infection

Lipidomic analyses of human amniotic fluid in spontaneous labor at term also revealed the presence of other lipid mediators derived from the lipoxygenase and epoxygenase pathways of PUFA metabolism (31). Lipoxygenases catalyze the hydroperoxidation of PUFA, which are further metabolized to leukotrienes, hydroxy PUFA, and Specialized Pro-resolving Mediators (SPMs) that comprise of resolvins, protectins, and maresins (56, 57). For example, conversion of arachidonic acid by 5-lipoxygenase produces some of the most potent lipid mediators of inflammation such as LTB_4_ and the cysteinyl leukotrienes, LTC_4_, LTD_4_, and LTE_4_ (58). While there is no significant difference in the levels of prostaglandins between patients in normal spontaneous labor and those with clinical chorioamnionitis (hence, prostaglandins cannot be accounted for this inflammatory condition), LTB_4_, the potent neutrophil chemotactic lipid mediator is almost exclusively present only in the amniotic fluid of patients with microbial invasion of the amniotic cavity and the associated clinical chorioamnionitis (59). Interestingly, neither of the other two clinical chorioamnionitis patient groups (i.e., without any detectable microbial invasion of the amniotic cavity but elevated cytokine levels (IL-6 > 2.6 ng/ml) or without any elevated IL-6 (<2.6 ng/ml), hence, no biochemical signatures of “inflammation”) have any detectable levels of LTB_4_ in the amniotic fluid (<0.015 nM). Hence, in the absence of microbial infection, LTB_4_, the most potent neutrophil chemoattractant, is also not responsible for eliciting inflammation in clinical chorioamnionitis.

## Regulation of Inflammation by Endogenous Anti-Inflammatory Mediators

A third pathway of PUFA metabolism is by cytochrome P450 dependent epoxygenases and monohydroxylases (60). Epoxygenases catalyze the epoxidation of PUFA by introducing an oxygen atom across the double bonds, whereas the monohydroxylases are responsible for the formation of ω-hydroxy fatty acids and drivers of catabolism. Epoxy-PUFA are designated as anti-inflammatory by virtue of their inhibition of NF-κB activation (61). These lipid mediators also exhibit significant physiological activity in reducing hypertension by regulating cardiovascular muscle tone (62), stimulation of endothelial cell proliferation to promote angiogenesis (63), as well as antipyresis (64).

Epoxygenase pathway metabolites are also major constituents of human amniotic fluid fatty acyl lipidome (31). Some of the epoxy-PUFA are about 3-fold more abundant than PGE_2_ in the amniotic fluid of patients in normal spontaneous labor [median: 65 nM for PGE_2_ vs. 200 nM for 11(12)-EpETrE at term in spontaneous labor]. Considering the established anti-inflammatory properties of the epoxy-PUFA [including antipyresis (64)], are these lipid mediators endogenous regulators that curtail the inflammatory propensity of PGE_2_ under normal physiological conditions? In other words, is PGE_2_ inflammatory only in the absence of “pressure” from anti-inflammatory lipid mediators such as epoxy-PUFA? If so, this would certainly explain the absence of clinical symptoms of inflammation in spontaneous labor at term, despite strong induction of COX-2 expression and super saturating (to the EP receptors) concentrations of PGE_2_ in the amniotic fluid. This idea gains further momentum from the fact that epoxy-PUFA [as well as the anti-inflammatory cytokine IL-10 (36)] are at significantly lower levels in amniotic fluid of patients in clinical chorioamnionitis without microbial infection (regardless of intra-amniotic inflammatory cytokine, IL-6, levels) compared to those in normal spontaneous labor (48). Thus, not only the high levels of epoxy-PUFA are associated with the absence of inflammation in the presence of receptor-saturating concentrations of PGE_2_ but also their absence (or significantly diminished levels) results in the manifestation of inflammation by PGE_2_. Another noteworthy example of a coordinated physiology between COX-2 and epoxygenases is the constitutive expression of COX-2 in renal medullary and cortical tissues without inherent inflammation. Renal cortical and medullary tissues also express PUFA epoxygenases and the biological activities of both enzymes are essential for the regulation of salt-sensitive hypertension (65). In essence, inductive expression of COX-2 and the biosynthesis of PGE_2_ do not manifest in clinical or biochemical signs of inflammation by default in the absence of injury, microbial or otherwise.

## A New Paradigm

This concept is a significant departure from the decades-old consensus because COX-2 induction and PGE_2_ biosynthesis always coincide with inflammation leading to the implicit association with assumed causality (8, 16). While anti-inflammatory properties also were attributed to COX-2, its role is in the resolution of inflammation rather than its inhibition (6, 15, 66). Indeed, when considered in isolation, COX-2 induction and elevated PGE_2_ levels do lead to inflammation. Even when there are no signs of inflammation at the behest of COX-2 expression (e.g., constitutive expression in several tissues) and PGE_2_ biosynthesis (e.g., in intestinal epithelium), the prevailing assumption has been that “an underlying sub-clinical inflammation” is at work. This assumption is partly due to limited information on the relative concentrations of the pro- and anti-inflammatory lipid mediators at the site of action in continuously irrigated tissues by circulation (virtually every tissue with possible exception of brain and privileged spaces like amniotic cavity). Besides, the local lipid mediator concentrations can be high at the site of their biosynthesis but are significantly diluted and/or metabolized by the time they are measured in circulation, imposing a practical limitation to glean appropriate physiological significance based on the circulating levels of these lipid mediators. Otherwise, examples COX-2 expression and PGE_2_ biosynthesis without inflammation are abound in cardiovascular, renal, pulmonary, musculoskeletal, and neuronal physiology (*vide supra*). While development of this new paradigm is based on PUFA-derived lipid mediators in the context of human parturition, its general applicability can be gleaned from similar paradoxical observations in cytokine literature. As mentioned above, increase in the intra-amniotic cytokine (both pro- and anti-inflammatory) levels coincide with spontaneous labor at term without any clinical symptoms of inflammation (35, 36). Interestingly, a decrease in the anti-inflammatory cytokine, IL-10, coincides with non-infectious and non-inflammatory cytokine-mediated clinical chorioamnionitis at term, similar to the decrease in anti-inflammatory epoxy PUFA (31, 36). Cytokines deemed to be pro-inflammatory exhibiting anti-inflammatory activities and vice versa depending on the context are plentifully documented (67–71). So are the paradoxical roles of lipid and protein “inflammatory” mediators in other physiological and pathophysiological processes (15, 41, 69, 72–78). Fortunately, the ability to measure mediator levels in the cloistered space of the amniotic cavity has offered a glimpse of their true local and real time concentrations in their native environment to assess their physiological role in target tissues.

**Figure 3:**
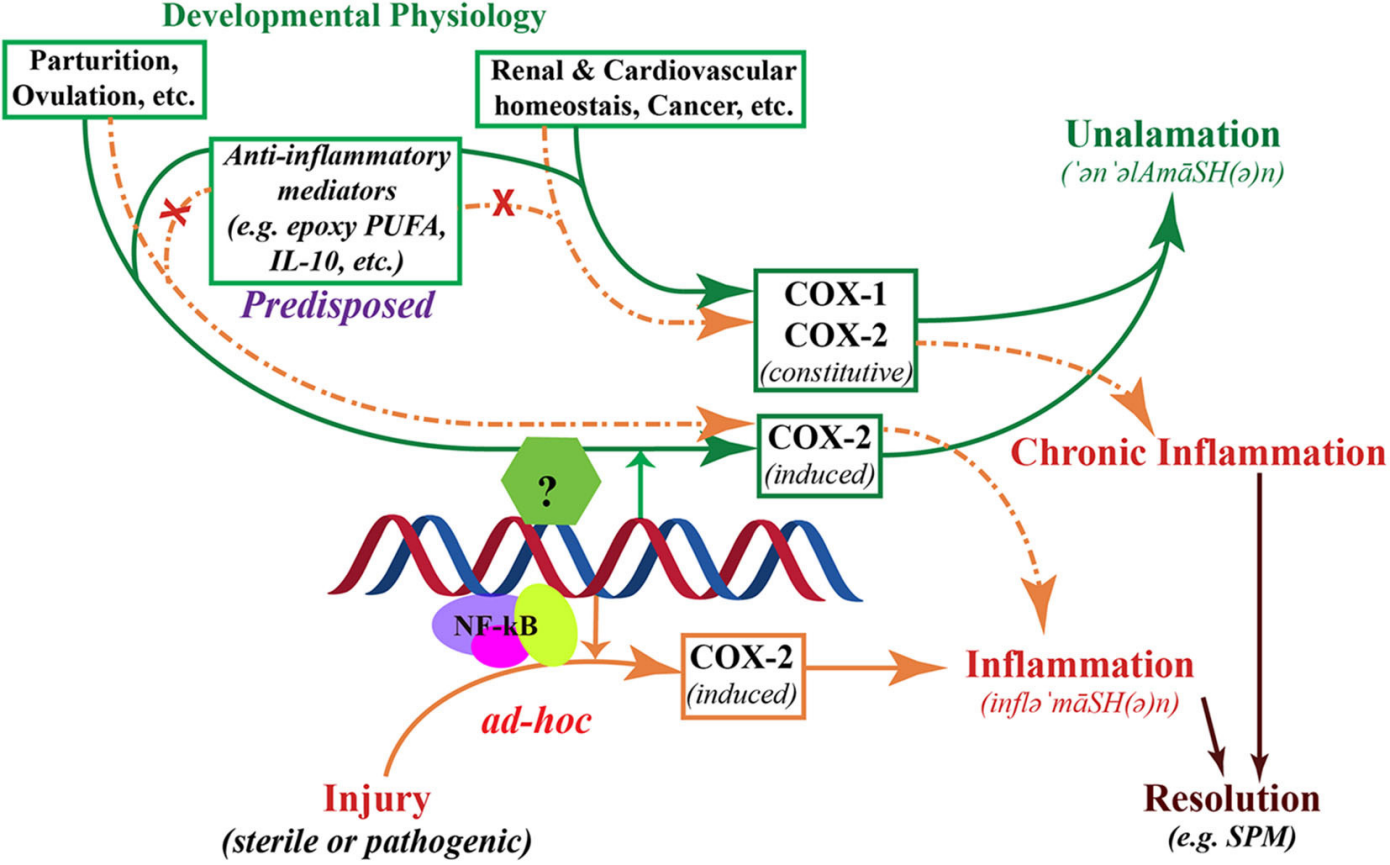
Schematic representation of the two divergent processes of inflammation and *unalamation*. Both physiological processes are mediated by the induction of cyclooxygenase (COX)-2, however, in *unalamation* the constitutive enzyme COX-1 and the induction or constitutive expression of COX-2 are associated with coordinated activation of anti-inflammatory lipid mediator biosynthesis thereby preventing the manifestation of clinical symptoms of inflammation (green arrows). When COX-2 is induced in response to injury, absence of such coordinated anti-inflammatory lipid mediator biosynthesis leads to clinical symptoms of inflammation (solid brown arrows). Such inflammation is implicitly resolved, but not inhibited, by Specialized Pro-resolving Mediators. Failure of anti-inflammatory predisposition in *unalamation* results in the manifestation of inflammation, indistinguishable from that elicited by injury (broken brown arrows). Failure of anti-inflammatory control in *unalamation* driven by induced COX-2 is akin to acute inflammation. Similar failure of *unalamation* by constitutively expressed COX-1 and/or -2-driven, on the other hand, could result in chronicity of inflammation due to the continued biosynthesis of “inflammatory” mediators by the constitutive enzymes. In either case, inhibition of COXs by non-steroidal anti-inflammatory drugs (NSAIDs), instead of augmenting the anti-inflammatory predisposition to curtail inflammation due to deranged *unalamation*, could result in adverse physiology.

Therefore, “inflammatory” lipid mediator biosynthesis and the associated enzyme expression in response to infection or injury is fundamentally *different* from the same biochemical pathway activated to drive a normal physiological process such as parturition, renal, cardiovascular or gastrointestinal homeostasis. While some of the cardinal signs of inflammation (such as increased blood flow and tissue remodeling) and the biosynthesis of mediators are shared between the two physiological responses, they are not the same to band them together as “inflammation” (a corollary to this distinction is “apoptosis” and “necrosis”). Physiological processes such as parturition utilize the same lipid mediators (e.g., PGE_2_) to induce appropriate changes in tissues and some clinical symptoms may appear to be similar to an “inflammatory” event. However, in a homeostatic physiological process, such changes ensue with a predisposed mechanism to prevent progress into inflammation, as opposed to a reactionary response to injury or infection (i.e., inflammatory). In other words, the initiation of a physiological process, where “inflammatory” mediators are involved, ensues with a pre-programmed (i.e., predisposed) anti-inflammatory mechanism set in place (Figure 3). This is essentially an orchestrated physiological program that is immunologically silent. When this 'program' fails or the tissue failed to “pre-program” this immunological silencing (*via* biosynthesis of anti-inflammatory mediators), the result is inflammation even in the absence of any infectious agents or injury. In case of injury (infectious or sterile), the very same lipid mediator biosynthesis is *ad hoc* and leads to an immunological response, which is inherently followed by an active resolution phase (3). Thus, the same lipid mediators participate in two distinct physiological processes, one with immunologically silent utilization of lipid mediators to aid a homeostatic process and the second that elicits an immunological response, i.e., inflammation, with pathological consequences. Classical induction or rescue experiments (both *in vitro* and *in vivo*), where COX-2 is induced experimentally or PGE_2_ is added exogenously, to establish their role in inflammation inevitably result in inflammation because of the lack of such pre-ordained “anti-inflammatory” control.

New terminology is warranted to distinguish these two physiological processes. I propose the name “*Unalamation*” ('*ən'əlAmāSH(ə)n*) for the immunologically silent participation of lipid mediators in a physiological process in contrast to “inflammation” (*inflə*'*māSH(ə)n*), where the participating lipid (and protein) mediators may be same but elicit a pathophysiological response. The name “*Unalamation”* is derived from “*unalam”* ('*ən'əl'əm)*, a Telugu word with Sanskrit origin for the mythical fire that regulates the burning of food in stomach and believed to help sustain the physiological processes. Distinguishing features of these two physiological processes are summarized in the Table 1.

**Table 1:** Defining features of the two physiological phenomena, “inflammation” and “*unalamation*.”

**Inflammation (*infle'māSH(e)n*)**	***Unalamation* (*'en'elAmāSH(e)n*)**
*A physiological host defense mechanism against foreign substances, pathogens, and physical injury*	*A non-inflammatory, homeostatic, developmental, physiological response of tissues to mediators that are otherwise known to induce/mediate inflammation*
*An ad hoc immunological response to tissue injury elicited by lipid and protein mediators*	*A pre-disposed homeostatic process that uses the same lipid mediators but does not elicit immunological response*
*Specialized pro-resolving mediators help mitigate damage following immune response*	*Endogenous anti-inflammatory lipid mediators steer mediators away from elicitation of inflammatory or immune response*
Mediators: Prostaglandins, leukotrienes, cytokines, and chemokines	Mediators: Prostaglandins, epoxy-PUFA, and anti-inflammatory cytokines

## Implications and Future Directions

Implications of this new concept are many-fold. First, the concept of *Unalamation* helps us understand the constitutive expression of COX-2 (the so-called “inflammatory” enzyme) in tissues such as brain, kidneys, small intestine, etc., as an enzyme participating in normal non-pathologic and non-immunogenic physiology. It also becomes imperative that constitutive COX-2 expression serves a “physiological” purpose for the survival and growth of tumor tissues (e.g., PGE_2_ is pro-angiogenic). Second, even the induction of COX-2 in developmental physiological context is non-inflammatory and its inhibition could be detrimental (e.g., interference of NSAIDs in parturition). Third, should the induction of COX-2 be exclusively inflammatory, highly selective and specific inhibitors of COX-2 would have been more effective non-steroidal anti-inflammatory drugs compared to less selective ones. Indeed, the most effective (yet, still toxic!) non-steroidal anti-inflammatory drugs in use are non-exclusive COX-2 inhibitors and the highly selective COX-2 inhibitors are yet to exhibit preferable pharmacology (79–81). Fourth, chronic inflammation could result either from impaired *Unalamation* as a result of failed anti-inflammatory pre-programming or insufficient resolution of inflammation following an injury. NSAIDs that do not discriminate inflammation verses *Unalamation* (virtually all NSAIDs in current use) are blunt pharmacological agents. Regular use of NSAIDs to treat chronic inflammatory conditions impedes *Unalamation* and results in adverse pharmacology. Efforts to distinguish these two physiological processes are vital to mitigate the collateral damage from inflammation and sustain the role of lipid mediators in non-inflammatory physiology through *Unalamation*. Finally, because of the similarities in the biochemical pathways that participate in *Unalamation* and inflammation, focusing on resolution physiology, that is associated with inflammation, is perhaps a more promising approach to mitigate inflammation-driven pathophysiology.

Here are a few outstanding questions that could be answered with further research to test this novel hypothesis:

- *Unalamation* utilizes the same mediators that can lead to inflammation and inhibition of inflammation. What are other mediators beyond Polyunsaturated Fatty Acid-derived lipids and IL-10 that participate in *Unalamation*?
- What is the balance of “inflammatory” vs. “anti-inflammatory” mediators required for *Unalamation* to maintain homeostasis?
- Inflammation is essential to protect the organism from infection/injury and non-steroidal anti-inflammatory drugs are vital to control acute inflammation. However, their use to treat chronic inflammation can negatively affect *Unalamation*. Can chronic inflammation be contained without negatively influencing *Unalamation*? Are Specialized Pro-resolution Mediators (SPMs) derived from ω-3 polyunsaturated fatty acids or similar counterparts in protein mediators the solution to mitigate chronic inflammation while sparing *Unalamation*?
- Antioxidants have been implicated in the regulation of chronic inflammatory conditions. Does dietary antioxidant supplementation indirectly assist *Unalamation* without interfering with inflammatory defenses against injury?
- *Unalamation* is a novel concept promulgated from observations in the unique physiology of parturition. While this concept is supported with inferences from renal, cardiovascular, and neuronal physiology, a challenge remains to demonstrate, by direct measurement, the same local balance of “inflammatory” and anti-inflammatory mediators in the face of constant irrigation of tissues by circulation.
- NF-κB activation plays a central role in inflammatory response to injury and infection by inducing COX-2 as well as the release of inflammatory cytokines. Is COX-2 induction in *Unalamation* independent of NF-κB activation? If not, what are the upstream regulatory mechanisms that guide COX-2 induction toward *Unalamation*?

## Data Availability Statement

All datasets presented in this study are included in the article/supplementary material.

## Author Contributions

KRM conceived the idea, formulated the theory, wrote the manuscript, and drew the illustrations.

## Conflict of Interest

The author declares that the research was conducted in the absence of any commercial or financial relationships that could be construed as a potential conflict of interest.

## Footnotes

^1^Mediators that participate in inflammation are by no means limited to PUFA metabolites. However, this new paradigm is based on inferences from the available human *in vivo* data on lipid mediators at the site of inflammation rather than *in vitro* or experimental model studies.
